# Target-responsive DNA-capped nanocontainer used for fabricating universal detector and performing logic operations

**DOI:** 10.1093/nar/gku858

**Published:** 2014-09-23

**Authors:** Li Wu, Jinsong Ren, Xiaogang Qu

**Affiliations:** 1 Laboratory of Chemical Biology, Division of Biological Inorganic Chemistry, State Key laboratory of Rare Earth Resource Utilization, Changchun Institute of Applied Chemistry, Chinese Academy of Sciences, Changchun, Jilin 130022, China; 2 University of Chinese Academy of Sciences, Chinese Academy of Sciences, Beijing 100039, China

## Abstract

Nucleic acids have become a powerful tool in nanotechnology because of their controllable diverse conformational transitions and adaptable higher-order nanostructure. Using single-stranded DNA probes as the pore-caps for various target recognition, here we present an ultrasensitive universal electrochemical detection system based on graphene and mesoporous silica, and achieve sensitivity with all of the major classes of analytes and simultaneously realize DNA logic gate operations. The concept is based on the locking of the pores and preventing the signal-reporter molecules from escape by target-induced the conformational change of the tailored DNA caps. The coupling of ‘waking up’ gatekeeper with highly specific biochemical recognition is an innovative strategy for the detection of various targets, able to compete with classical methods which need expensive instrumentation and sophisticated experimental operations. The present study has introduced a new electrochemical signal amplification concept and also adds a new dimension to the function of graphene-mesoporous materials hybrids as multifunctional nanoscale logic devices. More importantly, the development of this approach would spur further advances in important areas, such as point-of-care diagnostics or detection of specific biological contaminations, and hold promise for use in field analysis.

## INTRODUCTION

The unique material properties of nucleic acids have made them attractive polymer building materials for nanodevices and nanostructures due to their ability for self-recognition and self-assembly. Nucleic acids are prone to structural polymorphism: in addition to the well-known double helix, a number of alternative structures may be formed by designing their primary sequence. Single-stranded RNA/DNA enzymes and aptamers with a particular sequence, which were selected by the random screening methods, can exert their activities by forming a specific steric structure with intramolecular hydrogen bonding, stacking interactions, electrostatic interactions and metal coordination (1,2). These specific structures are the bases for the enormous potential of DNA-based devices in the fields of nanobiotechnology and biomedical technology. The targets of these single stranded DNAs can range from metal ions and small organic molecules to biomolecules, and even viruses or cells (3), making functional DNA a general platform for recognizing a broad range of targets (4) and fabricating molecular computing devices (molecular switches, logical units and programmable molecular systems for massively parallel computing) (5). Although these DNA nanodevices are promising, how to make them to perform more sophisticated functions in one system remains a big challenge in this field.

Here, we described the use of single-stranded DNA probes as a biomolecule-responsive cap system for graphene@mesoporous silica (MSGNs), and demonstrated the operability of this system with intelligent on-demand molecular transport for universal electrochemical detector fabrication and logic gate operations. The development of universal sensors that can detect a broad range of different targets has aroused much research interest, because such versatile platforms offer a single solution for tests that have traditionally required the use of a series of different types of instrumentation. Despite their great promise and many years of investigation, very few universal detection systems have been developed (6–10) and most do not show sufficient sensitivity for direct sample analysis or clinical use due to the lack of an amplification mechanism (11,12). The fabrication of efficient analysis methods with ultrahigh sensitivity, which are superior to those currently available, will fulfill unmet needs in scientific research, environmental monitoring, medical diagnosis and point-of-care testing.

Electrochemical assays of bioanalytes have attracted particular attention for their high sensitivity, simplicity, easy miniaturization and low-cost fashion through the use of compact instrumentation (13–16). The sensitivity of electrochemical sensors is critically dependent on the signal amplitude corresponding to a single target recognition event. Encapsulating numerous signal-generating molecules within a nanoparticle host is one of the promising strategies to dramatically increase the number of tags per binding event and achieve enormous signal amplification (17,18). Martinez-Manez *et* *al*. carried out the pioneering research pilot in designing gated materials, such as mesoporous silica, capable of responding specifically to a certain target molecule as a suitable method for developing new protocols for sensing applications (19–21). The advantage of the approach is the potential existence of amplification feature: the presence of a few analyte molecules may induce the inhibition or the release of a relatively high amount of entrapped dye. Given the possible use of different porous supports, diverse guest-selective gate-like systems and a wide range of indicator dyes, this strategy displays enormous potential and arouses much research interest for the development of novel signaling systems. In the past few years, this conceptually new protocols has been used for fluorescent or colorimetric detection of small molecules, metal ions, proteins and oligonucleotides, design of logic gate devices (22–28). As a new concept for signal amplification in electrochemical biosensor fabrication, the ideal porous materials could be served as the best candidate for the encapsulation of a large amount of redox-active molecules, which provides electronic amplification and thus facilitates high sensitivity readout: hundreds of electrons can result from each bimolecular binding event (29,30). To date, however, there has been few examples of pore-closing protocols based-on gated materials, such as mesoporous silica nanoparticles (MSNs), for electrochemical signal amplification, presumably because the poor conductivity of MSNs may severely cripple the performance of biosensor. As a one-atom-thick planar sheet of sp^2^-bonded carbon atoms, graphene owns unusual electronic properties, such as ballistic electron transport along with highly electrical conductivity (31–33). The unique physicochemical property suggests that it has great potential for providing new approaches and critical improvements in the field of electrochemistry (34,35). Significant progress has been made for the utilization of graphene in electrochemical biosensor fabrication, such as detection of DNA and metal ion, protein and pathogen, design of cell/bacterial nanodevice (36–40). However, multifunctional hybrid materials, by taking advantages of both the superior of graphene and the functional materials, remain largely unexplored in this field.

**Scheme 1.**
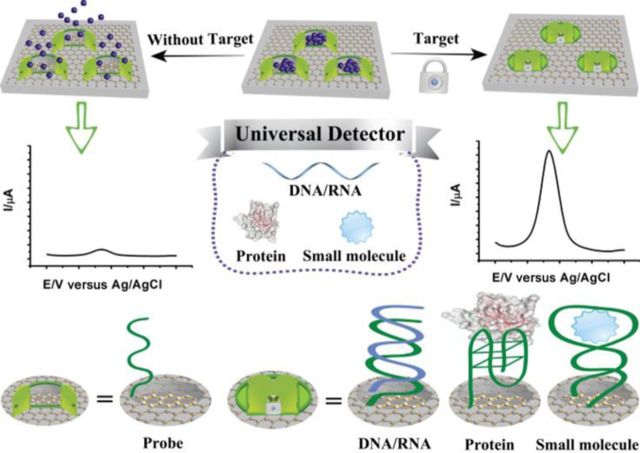
Schematic illustration of the TRE electrochemical biosensor for testing. In TRE detection, small molecules, nucleic acids and proteins are all detectable as each one cause conformation changes of single-strand probe DNA and a large amount of redox-active molecules are encapsulated, achieving an increase of current. It should be noted that the illustration is only a graphic presentation and does not represent the scale of each component and the precise way that DNA binds onto MSGNs.

Inspired by these insights, here we introduced a new approach to electrochemical detection, the target-responsive encapsulation assay (TRE) based on MSGNs, which achieves ultrahigh sensitivity with all of the major classes of biologically relevant molecules. For MSGNs, various functions can be incorporated into a single hybridized nanoparticle, which is designed for simultaneous signal probe encapsulation and accelerating electron transfer, without losing the individual property of each component. The principle that underlies the TRE is illustrated in Scheme 1. Single-stranded DNA probe is firstly conjugated to the MSGNs, serving as the intelligent ‘gatekeeper’ and switches its conformation in response to the corresponding target. The sensing mechanism relies on the strong signal amplification: the aqueous solution containing electro-active molecules (methylene blue, denoted as MB or ferrocene, denoted as Fc) can flow into the inner cavity of MSGNs by stirring, thus MSGNs are loaded with a large amount of electro-active molecules, which are only caged upon analyte-induced closing of the pores and generating measurable ‘off-on’ current. Furthermore, using MSGNs as a versatile matrix which can incorporate various redox-active molecules into the mesopores, simultaneous evaluation of different targets can also be realized in the present study. The new concept for developing this simple, label-free, reliable, highly sensitive and selective electrochemical turn-on strategy can also be utilized to design the ‘AND’, ‘OR’, ‘NOR’ and ‘INHIBIT’ logic gates using different metal ions as inputs and the current generated by both of MB and Fc as outputs.

**Figure 1.**
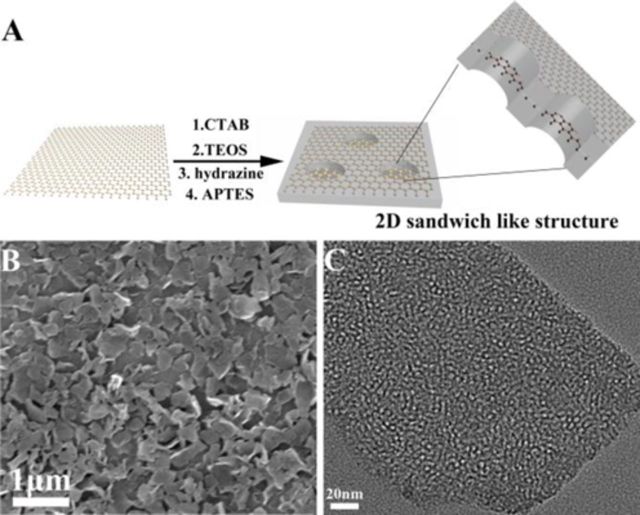
(**A**) Scheme for fabrication of MSGNs: (1) adsorption and assembly of CTAB on the surface of graphene oxide; (2) hydrolysis of TEOS; (3) the reduction of graphene oxide by hydrazine; (4) conjugation of amino groups by APTES. Typical SEM (**B**) and TEM (**C**) images of MSGNs with sizes from 100 nm to several micrometers and have a mesoporous structure.

**Figure 2.**
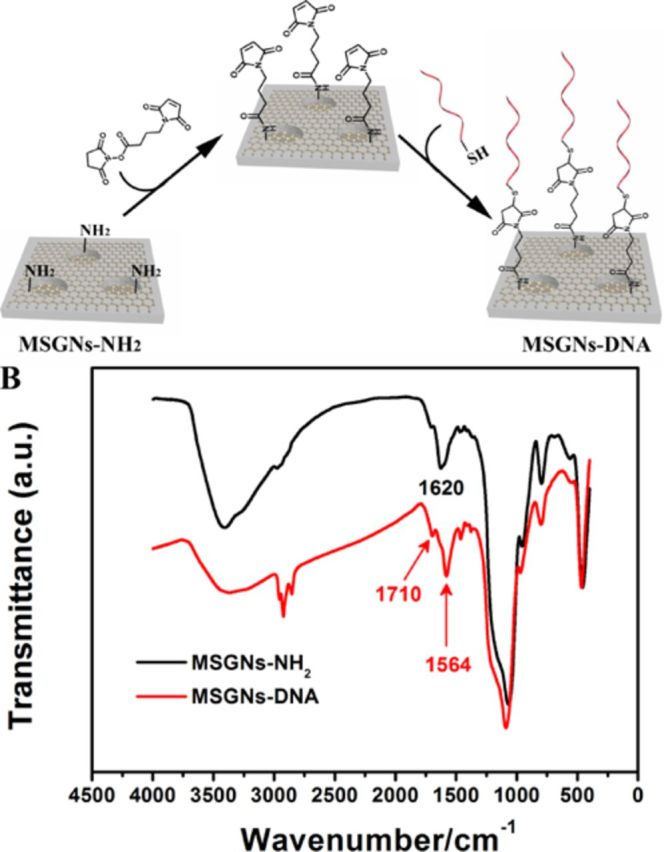
(**A**) Schematic illustration of surface immobilization of probe DNA on the MSGNs. (**B**) The FT-IR spectra of MSGNs-NH_2_ and MSGNs-DNA.

**Figure 3.**
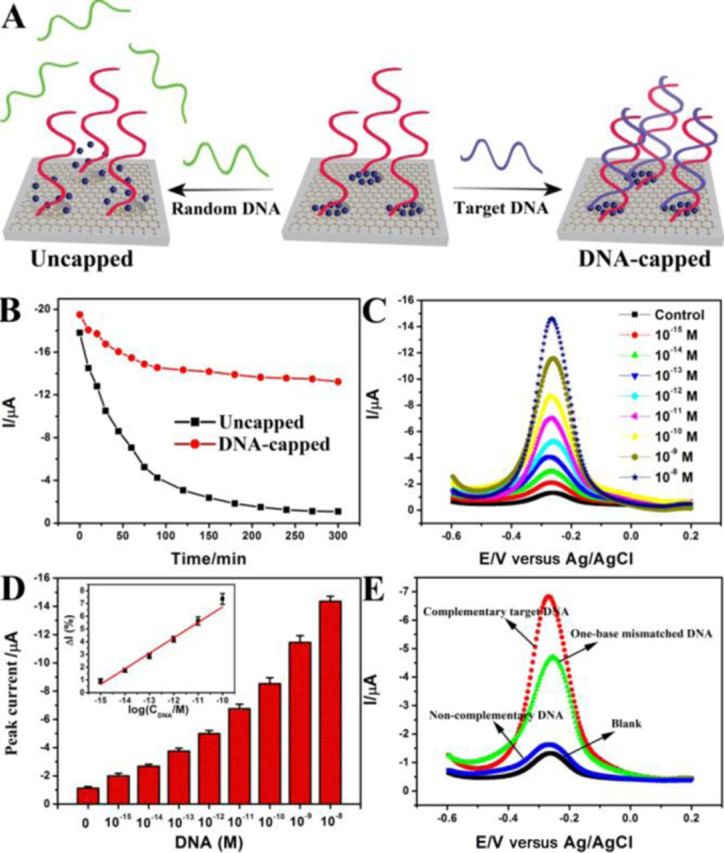
TRE nucleic acid detection. (**A**) Schematic representation of DNA detection by TRE. (**B**) Release profiles of methylene blue dye from probe DNA modified MSGNs with (red dot-line) or without (black dot-line) target DNA capping. (**C**) DPV signals for a DNA probe-modified sensor in response to different concentrations of target DNA: 0, 10^−15^ M, 10^−14^ M, 10^−13^ M, 10^−12^ M, 10^−11^ M, 10^−10^ M, 10^−9^ M, 10^−8^ M. (**D**) Concentration-dependent peak current signal for complementary DNA target. Inset: the linear plot. (**E**) The specificity of TRE sensor for the discrimination of target DNA, one-base mismatched DNA and noncomplementary random DNA, the concentration of DNA is 10 pM.

**Figure 4.**
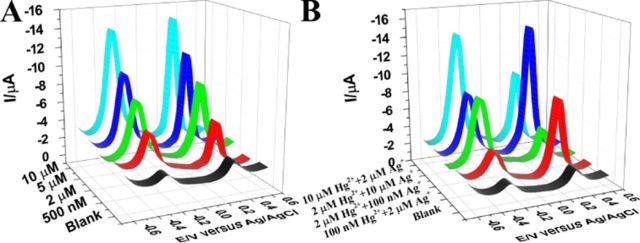
The DPVs corresponding to the multiplexed analysis of Hg^2+^ and Ag^+^ by MSGNs^MB^-DNA_Hg_^2+^ and MSGNs^Fc^-DNA_Ag_^+^: (**A**) upon interaction with the mixture of the same concentration of Hg^2+^ and Ag^+^, 500 nM, 2 μM, 5 μM, 10 μM. (**B**) Upon interaction with the mixture of the different concentration of Hg^2+^ and Ag^+^, 100 nM Hg^2+^ and 2 μM Ag^+^, 2 μM Hg^2+^ and 100 nM Ag^+^, 2 μM Hg^2+^ and 10 μM Ag^+^, 10 μM Hg^2+^ and 2 μM Ag^+^.

**Figure 5.**
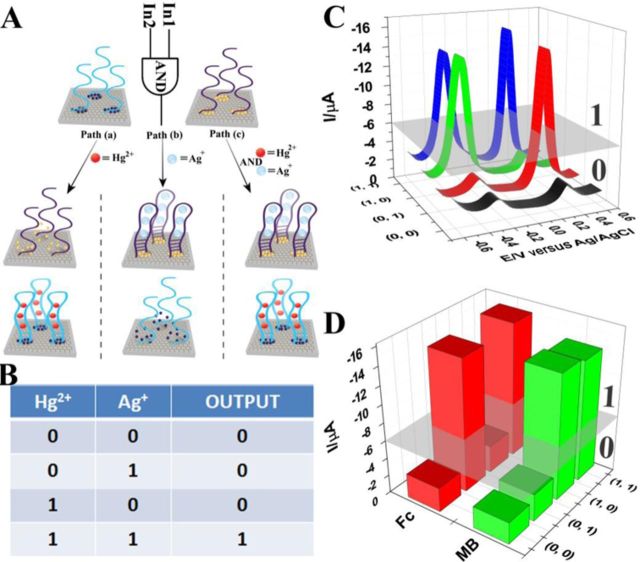
(**A**) TRE-based AND gate using MSGNs^MB^-DNA_Hg_^2+^ and MSGNs^Fc^-DNA_Ag_^+^; (**B**) truth table for AND gate; (**C**) DPVs of a TRE-based AND gate with different combinations of the input: (0,0) no input, (0,1) AND-input-1 (Ag^+^, 10 μM), (1,0) AND-input-2 (Hg^2+^, 10 μM), (1,1) AND-input-1 (Ag^+^, 10 μM) and AND-input-2 (Hg^2+^, 10 μM); (**D**) relative current intensities for the AND logic gate.

**Figure 6.**
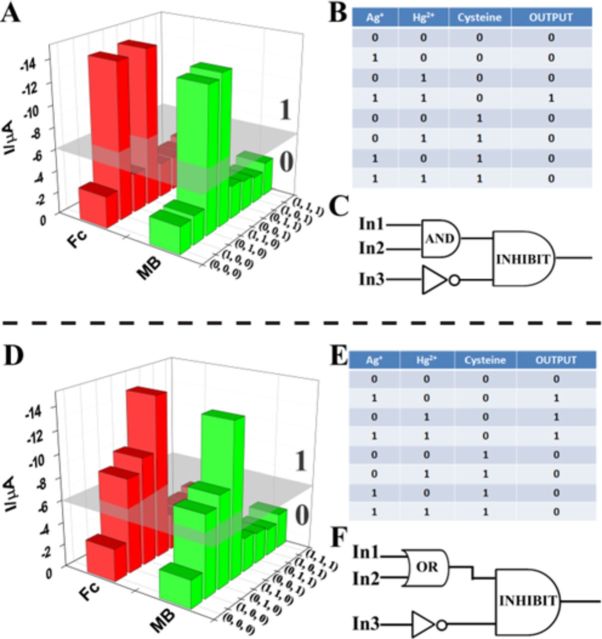
Relative current intensities for the Boolean logic functions based on TRE and eight combinations of three inputs: In1 = Ag^+^, In2 = Hg^2+^, In3 = Cysteine, (**A**) [Output = (A AND B) INHIBIT C], (**D**) [Output = (A OR B) INHIBIT C]. Truth table (**B**) and (**E**) and logic circuitry for the integrated logic system (**C**) and (**F**).

## MATERIALS AND METHODS

### Materials

Graphite was purchased from Sinopharm Chemical Reagent (Shanghai, China). Tetraethylorthosilicate (TEOS), (3-aminopropyl) trimethoxysilane (APTES) and Tris-(2-carbozyethyl) phosphine hydrochloride (TCEP) were purchased from Sigma-Aldrich and used as received. 4-Maleimidobutyric acid N-hydroxysuccinimide ester (GMBS) (97%) was obtained from Acros Organics. N–cetyltrimethylammonium bromide (CTAB) was obtained from Alfa Aesar. Hydrazine (85%) and hydrochloric acid were purchased from Beijing Chemicals Inc. (Beijing, China). The NTPs (adenosine triphosphate (ATP), GTP, CTP and UTP) and the oligonucleotide used in this paper were offered by Biotechnology Inc. (Shanghai, China). All other reagents were all of analytical reagent grade and used as received. All aqueous solutions were prepared with nanopure water (18.2 MΩ cm, Milli-Q, Millipore).

The sequences of the DNA probes and targets used in this work:

26-mer-Linear-Probe: 5′-SH-(CH_2_)_6_-ACC AGG CGG CCG CAC ACG TCC TCC AT-3′
26-mer-Linear-target 0 mismatch: 5′-ATG GAG GAC GTG TGC GGC CGC CTG GT-3′
26-mer-Linear-target 1 mismatch: 5′-ATG GAG GAC GTG CGC GGC CGC CTG GT-3′
26-mer-Linear-noncomplementary target: 5′-AAA AAA AAA AAA AAA AAA AAA AAA AA-3′
Thrombin aptamer: 5′-SH-(CH_2_)_6_-GGT TGG TGT GGT TGG-3′
ATP aptamer: 5′-SH-(CH_2_)_6_-ACC TGG GGG AGT ATT GCG GAG GAA GG-3′
Mercury-specific oligonucleotide (MSO) probe: 5′-SH-(CH_2_)_6_-TGT TTC TTT CTT CCC CTT GTT TGT TTC A-3′
Silver-specific oligonucleotide (MSO) probe: 5′-SH-(CH_2_)_6_-CTC TCT TCT CTT CAA AAA ACA ACA CAA CAC AC-3′

### Apparatus and characterization

Transmission electron microscopic (TEM) images were recorded using a FEI TECNAI G2 20 high-resolution transmission electron microscope operating at 200 KV. Scanning electronic microscopy (SEM) images were obtained with a Hitachi S-4800 FE-SEM. FT-IR characterization was carried out on a BRUKE Vertex 70 FT-IR spectrometer. The samples were thoroughly ground with exhaustively dried KBr. X-ray photoelectron spectroscopy (XPS) spectra were obtained with an ESCALAB Thermal 250 instrument and monochromatic Mg-Ka (*E* = 1253.6 eV) was used for photoexcitation. N_2_ adsorption-desorption isotherms were recorded on a Micromeritics ASAP 2020M automated sorption analyzer. The samples were degassed at 150°C for 5 h. The specific surface areas were calculated from the adsorption data in the low pressure range using the Brunauer-Emmet-Teller (BET) model and pore size was determined following the Barrett-Joiner-Halenda (BJH) method. Electrochemical measurements were performed with a CHI 660B Electrochemistry Workstation (CHI, USA). A three-electrode setup was used with a common Ag/AgCl reference electrode, a glassy carbon working electrode and a Pt wire auxiliary electrode placed in the central buffer solution. The glassy carbon electrodes (GCE, *ø* = 3 mm, CHI) were first polished successively with 1.0, 0.3 and 0.05 μm alumina (Buhler) and sonicated for 3 min before modification.

### Preparation of MSGNs-NH_2_

Graphene oxide (GO) was synthesized from graphite by modified Hummers method (41). The procedure of synthesizing MSGNs was followed by the literature with some modification (42). Briefly, 5.8 ml the as synthesized GO (3.8 mg/ml) aqueous solution was added into 44.2-ml water containing 0.5-g CTAB and 20-mg NaOH, and then ultrasonically treated for 1 h. After magnetic stirring for 2 h at 40°C, tetraethylor-thosilicate (TEOS, 400 μl dissolved in 1.6-ml ethanol) was slowly added to the above mixture. After reaction for 12 h, 80 μl of hydrazine was additionally introduced into the above mixture, and then heated at 70°C for 5 h. The obtained product was centrifuged and washed with warm ethanol for three times. The product was then mixed with 200-μl APTES in 50-ml ethanol and stirred for 12 h at 80°C under reflux before centrifugation. Finally, the product was dispersed in 50-ml acetone stirred at 40°C for 24 h. The product was collected by centrifugation and washed by warm ethanol for three times. The product MSGNs-NH_2_ was washed and dissolved in the proper amount of water.

### TRE sensor fabrication

A bifunctional cross-linker GMBS was used to functionalize MSGNs-NH_2_ with DNA probe or aptamer, the procedure was according to the previous report (43). Briefly, MSGNs-NH_2_ (10 mg) was well suspended in a mixture solution of phosphate buffered saline (PBS) buffer (100-mM PBS, 150-mM NaCl, pH 7.3) and N,N-Dimethylformamide (DMF) (7:3) containing excess GMBS (4 mg) for several hours. The resulting particles were collected by centrifugation, extensively washed with DMF and PBS buffer and give rise to maleimide-modified MSGNs. Further functionalization of the DNA was performed by first incubating 20-nmol of DNA with three equivalent of TCEP to obtain free sulfhydryl groups. Then, the treated DNA was mixed with maleimide-modified MSGNs in conjugation buffer (100-mM PBS, 1-M NaCl, pH 7.3) and stirred overnight at room temperature. The MSGNs-DNA was recovered by centrifugation and washing with the conjugation buffer.

The MSGNs-DNA nanocarriers (10 mg) were then soaked in a solution containing redox-active molecules (1 mg) in PBS buffer (100 mM, pH 7.4) and stirred overnight to allow the guest molecules to diffuse into the pores of the MSGNs-DNA. The nanoparticles were then centrifuged and washed thoroughly with buffer to remove adsorbed molecules.

### Target detection

The above fabricated TRE detection platform (200 μg/ml, 20 μl) was mixed with the target solutions (1 μl) with different concentrations, respectively. For synthetic target DNA, sensors were incubated for 2.5 h at room temperature with a phosphate buffer solution (25 mM, pH 7.0) containing 25-mM NaCl, 10-mM MgCl_2_ and different concentrations of the target. For ATP detection, sensors were incubated for 2.5 h at room temperature with a phosphate buffer solution (25 mM, pH 7.0) containing 25-mM NaCl and different concentrations of ATP. For thrombin detection, sensors were incubated for 2.5 h at room temperature with a phosphate buffer solution (25 mM, pH 7.0) containing 25-mM NaCl and different concentrations of thrombin. For Ag^+^ and Hg^2+^ detection, sensors were incubated for 2.5 h at room temperature with a HEPES buffer solution (20 mM, pH 7.0) containing 100 mM NaNO_3_ and different concentrations of metal ions. All the samples were then centrifuged at 12 000 rpm for 10 min, and the precipitates were collected and washed with buffer. In a typical test, chemicals were redispersed in 20-μl buffer and subsequently dropped on the pretreated GCE and dried at room temperature. The surface was then washed by PBS solution and stored under atmosphere prior to use. For electrochemical detection, the modified electrodes were scanned in a common running PBS buffer (25 mM, 25-mM NaCl). Differential pulse voltammetry (DPV) signals were obtained with a potential step of 5 mV, pulse amplitude of 50 mV, pulse width of 50 ms and a pulse period of 200 ms.

## RESULTS AND DISCUSSION

### MSGNs characterization

As shown in Figures 1A, the 2D sandwich like MSGNs-NH_2_ (both side of graphene was covered with mesoporous silica) was firstly prepared via soft template-assisted reducing process (42). In this procedure, the cationic surfactant CTAB was chosen to electrostatically adsorb and self-assemble onto the surface of highly negatively charged GO in alkaline solution. Upon hydrazine reduction treatment and the soft-template removing, the MSGNs products were successfully collected with mesoporous silica around the surface of single-layer GO. The surface of MSGNs was then functionalized with amine groups by treatment with 3-APTES to afford MSGNs-NH_2_. As shown in Figure 1B, many free-standing nanosheets with the sizes from 100 nm to several micrometers were observed in SEM image. TEM image (Figure 1C) further demonstrated that these nanosheets possessed numerous mesopores and it was obviously that the mesoporous silica was homogenously coated on the surface of graphene sheets (42). XPS is an efficient technique to analyze chemical state information of elements, which provides quantitative information about the type and extent of surface functionalization on the MSGNs-NH_2_ nanocomposites (44). XPS spectra of MSGNs-NH_2_ (Supplementary Figure S1A) clearly indicated the presence of silicon, carbon, nitrogen and oxygen. Reduction of oxygen functional groups in MSGNs was also confirmed by XPS of samples of GO (Supplementary Figure S1B and 1C). It is well-known that GO is an electrically insulating material, which arises from the presence of a wide range of oxygen functional groups on their basal planes, such as epoxide, hydroxyl, carboxyl and carbonyl groups (45). In comparison to the C1s spectrum of the GO, peaks assigned to oxygen-containing functional groups in MSGNs-NH_2_ were significantly decreased after reduction (Supplementary Figure S1B). N_2_ adsorption–desorption isotherms of MSGNs-NH_2_ showed a typical Type IV curve with a specific surface area of 476 m^2^/g, average pore diameter of 2.42 nm and a narrow pore distribution (Supplementary Figure S2).

### Sensor fabrication

Supplementary Figure S3 showed typical cyclic voltammograms (CVs) of K_3_[Fe(CN)_6_] on different surfaces. A pair of enhanced oxidation and reduction peaks was observed at the MSGNs-NH_2_ modified GCE compared with the mesoporous silica modified GCE, which indicated that the component of graphene in MSGNs-NH_2_ could facilitate electron transfer due to its good conductivity and large surface area. The resulting MSGNs-NH_2_ was reacted with a bifunctional cross-linker 4-Maleimidobutyric acid N-hydroxysuccinimide (NHS) ester which contains an amine-reactive N-hydroxysuccinimide functional group (NHS ester) and a sulfhydryl (thiol)-reactive maleimide group (43). The maleimide-terminated MSGNs were subsequently conjugated with the thiol-modified DNA probe to produce MSGNs-DNA for the following analysis (Figure 2A) (43). The surface functionalization of MSGNs was monitored by Fourier transform infrared spectroscopy (FTIR) spectroscopy (Figure 2B). The emerging absorption band at around 1620 cm^−1^ in the sample MSGNs-NH_2_ can be assigned to N–H stretching of the amino groups. The efficient grafting of DNA onto mesoporous silica was validated by the appearance of the characteristic asymmetric stretching mode of the imidyl group (1710 cm^−1^) and the acrylamide vibrations (1564 cm^−1^) in the FTIR spectroscopy (43).

### Nucleic acid detection for Alzheimer's disease

Using DNA sequences correlated to Alzheimer's disease (AD) as the model, we examine the ability of TRE sensor to detect nucleic acids. AD is a neurodegenerative disorder which affects millions of people in the aging population worldwide (46). One genetic risk factor for the development of the disease is the presence of a mutation in the Apolipoprotein E (apo-E) gene (47). The apo-E gene is polymorphic and presents a dysfunctional allele (apo-E4) in which a thymine is replaced by a cytosine (48). The presence of this mutation is involved in the development of the disorder. The complementary target (wild-type) used in present systems corresponds to the non-mutated Apo-E gene and the target with one base mutation (mutant) corresponds to the dysfunctional allele (apo-E4) (49,50). The configuration of the TRE assay for DNA detection was shown in Figure 3A. The redox-active molecule MB was introduced to fill in the interior pores of MSGNs-DNA. Since the diameter of the B-form duplex DNA structure is 2.0 nm, the closing of the nanocontainer was achieved by the gate-like duplex DNA structure formation to gate the pore and thus prevent the MB molecules from escaping (51). A single target-DNA recognition event resulted in the entrapment of substantial number of MB, generating the amplified current-signal. Figure 3B showed the time dependence of the sensor response. The current (*I*) was reduced by 80% when the uncapped MSGNs-DNA was cultured in buffer for 150 min. Once treated with the complementary probe, a structural change of the probe DNA from single strand to double helix caused an encapsulation of MB, which led to an augment of current. The current change could directly reflect how much DNA was present in the solution (Figure 3C). The peak current of DPVs increased with increasing concentration of the target within a range that spanned seven orders of magnitude (Figure 3D). A semilogarithmic dependence was obtained between the change of current (Δ*I*%) and DNA concentration (Figure 3D inset). This assay could measure DNA down to ∼1 fM without the involvement of complex and costly target ampliﬁcation, which was intriguing because previous studies on developing universal detection systems had not been successful in achieving such a good sensitivity with this analyte class (7,9).

The specificity of the assay was further investigated by measuring DPVs. Relative to the fully complementary target, much smaller increase in current was observed for one-base mismatched DNA (62.32% of that generated by wild-type DNA target at the same concentration) and almost neglectable change was observed for non-complementary DNA (Figure 3E). The suitability of the sensor for the analysis of heterogeneous samples was assessed by carrying out DNA detection in 50% human serum and undiluted human serum (Supplementary Figure S4). No obviously changes were observed for TRE sensor performed in serum alone compared to those performed in buffer, indicating the sensor was stable in serum. Comparison of the electrochemical signals collected in the presence of complementary DNA in buffer, 50% human serum and undiluted human serum confirmed that successful detection could be achieved in these complex matrixes. With a high level of performance established for nucleic acids analytes, we investigated whether it could detect protein biomarkers and small molecules, thrombin and ATP were selected as model targets.

### Detection of thrombin

α-thrombin is a coagulation protein that has many effects on the coagulation cascade and its determination can be used to understand thrombosis and homeostasis processes. This protein, also known as coagulation factor II, is a trypsin-like serine protease protein that is encoded by the F2 gene in humans, acts as a serine protease that converts soluble fibrinogen into insoluble strands of fibrin (52,53). The thrombin aptamer is a well-characterized sequence that is known to fold into a G-quartet structure and bind thrombin at exosite I (54). Figure S5 represents the protein-detection method using TRE approach. A thiolated thrombin-binding aptamer was linked onto MSGNs-NH_2_ surface. In the absence of thrombin, the MSGNs were directly exposed to the external environment and the substrates could diffuse freely via the mesoporous silica layer, the current was low. The current was stored upon addition of thrombin as the protein bound immediately with its aptamer and forming a unimolecular DNA quadruplex (55) as the block layer on the surface of the MSGNs, which induced the entrapment of electrochemical signal generated molecules-MB within the inner pores of MSGNs (Supplementary Figure S5A). The current was clearly increased in accordance with the raised concentrations of thrombin, as exhibited in Supplementary Figure S5B and C. Evaluation of ΔI% with different thrombin concentrations showed that as low as 10-fM thrombin was clearly detectable (Supplementary Figure S5C inset). The unique structure of MSGNs played a key role in signal amplification and resulted in significantly decreased detection limit, which was better than the most of previous methods (56–59). Furthermore, the fabricated TRE sensor illustrated superior specificity for thrombin detection. As indicated in Supplementary Figure S5D and E, when treated with 1 nM of thrombin, a large increase in current was observed. While conversely, when treated with other nonspecific proteins, such as bovine serum albumin, lysozyme (Lys) and immunoglobulin G (IgG), the signal change was negligible, indicating the assay was selective for thrombin recognition.

### Detection of ATP

To examine the ability of our TRE assay to sense small molecules, we first choose ATP as a model molecule. ATP is a nucleoside triphosphate used in cells as a coenzyme, which is often called the ‘molecular unit of currency’ of intracellular energy transfer (60). The schematic illustration of the TRE assay for ATP detection is shown in Supplementary Figure S6A. The assay was carried out using the same strategy as described above for thrombin, with an ATP-specific aptamer as the receptor. Once challenged with ATP, a conformational change was happened for ATP binding aptamer conjugated on MSGNs surface from single strand to duplex and G-quartet hybrids (61), which successfully gated the pores of MSGNs and prevented the leakage of MB, generating the measurable current (Supplementary Figure S6A). As observed for thrombin, the concentration dependence of the signal change observed with the addition of ATP (Supplementary Figure S6B and C). The ATP concentrations as low as 10 pM could be detected (Supplementary Figure S6C inset), which was superior to the previously reported techniques for ATP assay (62–64). Under the same conditions, other nucleoside triphosphate, such as CTP, GTP and UTP did not produce striking current enhancement, suggesting that this sensor has a high selectivity for ATP (Supplementary Figure S6D and E).

### Multiple sensing capabilities for metal ions detection

The flexibility of the TRE assay allows for the analysis of multiple analytes. The extent of multiplexing that can be achieved with this approach is controlled by the TRE sensing-matrix design. These multiplexed devices use two or more target-specific DNA probes that, when interrogated together, they can convert the recognition of specific targets events into specific outputs (65,66). Here, two TRE detection platforms were designed for Hg^2+^ and Ag^+^, respectively: MSGNs^MB^-DNA_Hg_^2+^ (potential at −0.25 V versus Ag/AgCl) (Supplementary Figure S7A) and MSGNs^Fc^-DNA_Ag_^+^ (potential at +0.28 V versus Ag/AgCl) (Supplementary Figure S7B). Various ions can form stable complexes with nucleic acids by interacting with specific nucleotide bases (67). A rigid hairpin structure is formed in the presence of Hg^2+^ or Ag^+^ ions, in which the T or C residues of the spatially separated nucleotides are linked by the ions. As demonstrated in Supplementary Figure S7 C-H, both of the sensors showed predominant sensitivity and selectivity in sensing of metal ions. The results indicate that Hg^2+^ and Ag^+^ can both be analyzed to a detection limit of 1 nM. The successful selective analysis of Hg^2+^ and Ag^+^ made it possible for multiplexed analysis of the two ions by the mixture of these two types of TRE sensors (Figure 4). Each of the two sensing platforms in the mixture possessed its own ability to hybrid with its corresponding target with high specificity without interference.

### Logic gate operations

Besides using the TRE as electrochemical transducers for universal detector fabrication, we implemented them as electrochemical labels to follow logic operations using Hg^2+^- and Ag^+^-responded TRE as the model. Logic gates provide the functional units of computers, which are capable of performing Boolean logic (68,69). A DNA logic gate is a powerful computational device and has the ability to interact with biological and chemical environments due to its biocompatible and programmable sequence-specific recognition ability (70–76). Most of the DNA logic gates reported to date are mainly based on fluorescence (65,77–79), gel-based electrophoresis (80) or colorimetric outputs (81,82), which are laborious, time-consuming and unsuitable for directly detecting subtle structures. To this end, we designed reagentless, molecular logic gates based on TRE that produced electronic (electrochemical) signals as their outputs. They combined the advantages of graphene and mesoporous silica and target-induced DNA conformational change into logic operations.

First, we designed an AND logic gate using Hg^2+^ and Ag^+^ as inputs that operated through the ensemble of MSGNs^MB^-DNA_Hg_^2+^ and MSGNs^Fc^-DNA_Ag_^+^ (Figure 5A). With respect to the input, we defined the presence of Hg^2+^ or Ag^+^ as ‘1’ and their absence as ‘0’. As described above, the current maximum of MSGNs^MB^-DNA_Hg_^2+^ was centered at −0.25 V, while for MSGNs^Fc^-DNA_Ag_^+^, the current maximum was centered at +0.28 V. For output, herein we regarded a current intensity of −6 μA as the threshold value. When the current intensities of the DPVs at −0.25 V and +0.28 V were lower than the threshold value, the output was defined as ‘False’ output or ‘0’; whereas the output = ‘Ture’ output or ‘1’ when the intensities of the current at −0.25 V and +0.28 V were both high (>threshold current). When no metal ions were added, that was corresponding to 0/0 input, both of the probe DNA kept in the form of single stranded, generating a low current both at −0.25 V and +0.28 V (output = 0). When Ag^+^ was added alone (input = 0/1), only the current at +0.28 V was increased while the current at −0.25 V was below the threshold current, the output was still defined as ‘0’. The case was similar in the presence of Hg^2+^ alone (input = 1/0). Addition of both Hg^2+^ and Ag^+^ kept the current at −0.25 V and +0.28 V at a high level (output = 1). The pattern of chemistry inputs to electrochemical outputs successfully created the truth table for the ‘AND’ logic gate, as shown in Figure 5B. Figure 5C and D showed the electrochemical features of the TRE sensor in the presence of the different inputs and demonstrated the feasibility of using MSGNs in the logic gate design. The system presented here employs a double-electrochemical output channel instead of a single-electrochemical output channel, which would increase the accuracy of the system (83,84).

Similarly, an OR logic gate can be constructed using Hg^2+^ and Ag^+^ ions as inputs, and operating through the ensemble of MSGNs^MB^-DNA_Hg_^2+^/DNA_Ag_^+^ and MSGNs^Fc^- DNA_Hg_^2+^/DNA_Ag_^+^ (Supplementary Figure S8). The pores of MSGNs could be locked in two parallel target-probe interactions, thus realizing the OR logic gate. With no input (input = 0/0), none of the probe changed its conformation to gate the pores, the currents at −0.25 V and +0.28 V were both below the threshold value, and thus the output of the system was ‘0’. In the presence of either or both inputs (1/0, 0/1, 1/1), the pores was shut by either or both pathways. As a result, the TRE sensor exhibited a dramatic current increase, which was considered as output ‘1’ (Supplementary Figure S8B–D). According to previous reports, glutathion (GSH) or cysteine (Cys) can strongly react with mercury ions (85), the interaction between target GSH/Cys will disorder the T-Hg^2+^-T formation, making the double-stranded DNA to dissociate. Based on these principles, an INHIBIT logic gate could be created by employing Hg^2+^ ion as one input, the addition of Cys as another input, and the DPV signal was defined as output (Supplementary Figure S9). The addition of Cys (input = 0/1) could not lead to the significant current enhancement of the TRE sensor because duplex DNA was not formed. In the presence of both Hg^2+^ and Cys (input = 1/1), the chelating of mercury ions with Cys led to the dispersion of signal-reporter molecules, which turned off the electrochemical signal. Only the addition of Hg^2+^ (input = 1/0) could cause the high electrochemical signal (output = 1). By observing the current intensity both at −0.25 V and +0.28 V, much higher value was obtained for the presence of Hg^2+^ (input = 1/0) than those for inputs (0/0, 1/0, 1/1) (Supplementary Figure S9B–D). In addition, a NOR gate using GSH and Cys as inputs was also designed (Supplementary Figure S10A). When neither of the thiol agents GSH and Cys was added (input = 0/0), the output was 1, otherwise it was 0 (Supplementary Figure S10B–D).

The aforementioned examples indicated that the TRE sensors were capable to perform several types of logic operations. However, the major challenge in the logic systems is the possibility of assembling multicomponent/multifunctional logic circuitries. In the following set of representative experiments, we explored our logic system could be scaled up to perform networking systems. Eight combinations of different sets of three input signals were used to generate the equivalent output of the circuits (Figure 6).

## CONCLUSION

In summary, a universal electrochemical DNA detection system presented here combined the advantages of both graphene and mesoporous silica, and achieved high sensitivity for all the tested analytes (Supplementary Table S1) and performed complicated logic operations by using single-stranded DNA probes as pore-caps of MSGNs. This logic system was scaled up to perform networking systems. Eight combinations of different sets of three input signals were used to generate the equivalent output of the circuits. This bio-specific recognition based on graphene and mesoporous silica may provide new insights into point-of-care diagnostics or detection of specific biological contaminations.

## SUPPLEMENTARY DATA

Supplementary Data are available at NAR Online.

SUPPLEMENTARY DATA
