# Supplementary material for: Target-responsive DNA-capped nanocontainer used for fabricating universal detector and performing logic operations
Source: Nucleic Acids Res. 2014 Sep 23;42(21):e160. doi: 10.1093/nar/gku858 (PMC4245965; doi:10.1093/nar/gku858)
Supplement: SUPPLEMENTARY DATA [file supp_42_21_e160__index.html]

Target-responsive DNA-capped nanocontainer used for fabricating universal detector and performing logic operations — Target-responsive DNA-capped nanocontainer used for fabricating universal detector and performing logic operations — Target-responsive DNA-capped nanocontainer used for fabricating universal detector and performing logic operations — SUPPLEMENTARY DATA 

# Target-responsive DNA-capped nanocontainer used for fabricating universal detector and performing logic operations

## SUPPLEMENTARY DATA

**Files in this Data Supplement:**

- SUPPLEMENTARY DATA
