# Supplementary material for: Target-responsive DNA-capped nanocontainer used for fabricating universal detector and performing logic operations
Source: Nucleic Acids Res. 2014 Sep 23;42(21):e160. doi: 10.1093/nar/gku858 (PMC4245965; doi:10.1093/nar/gku858)
Supplement: SUPPLEMENTARY DATA [file supp_gku858_nar-02199-met-f-2014-File002.pdf]

SUPPLEMENTARY DATA

Target-Responsive DNA-capped Nanocontainer Used for Fabricating Universal Detector and Performing Logic Operations

*Li Wu,[1,2] Jinsong Ren[1] and Xiaogang Qu[1]**

[1] Laboratory of Chemical Biology, Division of Biological Inorganic Chemistry, State Key laboratory of Rare Earth Resource Utilization, Changchun Institute of Applied Chemistry, Chinese Academy of Sciences, Changchun, Jilin 130022, China

[2] University of Chinese Academy of Sciences, Chinese Academy of Sciences, Beijing, 100039, China

Supporting Figures:

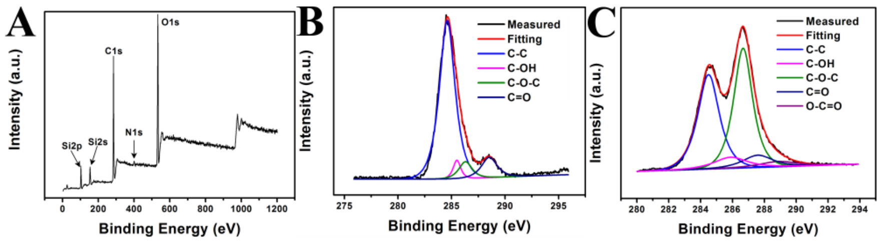

Figure S1. XPS profiles of (A) MSGNs, (B) carbon 1s of MSGNs and (C) carbon 1s of graphene oxide.

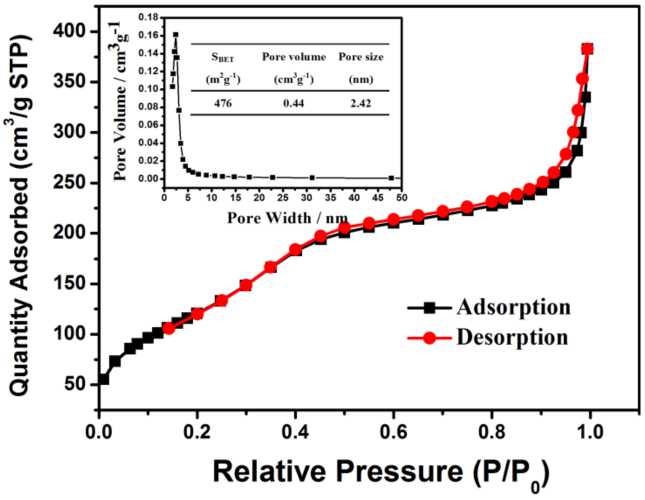

Figure S2. BET nitrogen adsorption−desorption isotherm and BJH pore size distribution curve (inset).

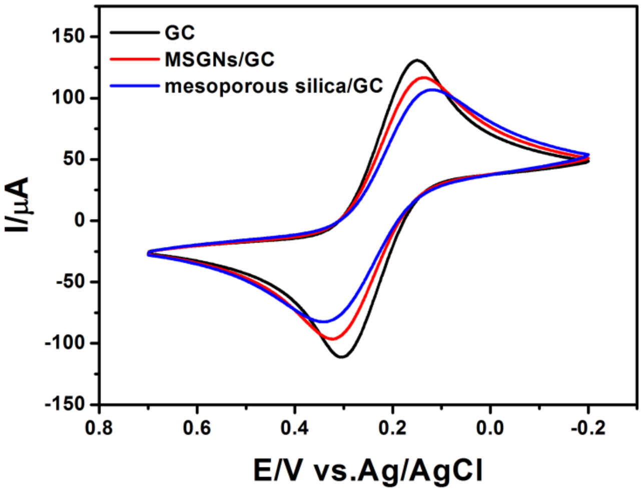

Figure S3. Cyclic voltammograms of bare glassy carbon (GC) electrode, MSGNs and mesoporous silica modified electrode in 10 mM $K_3[Fe(CN)_6]$ containing 0.1M KCl.

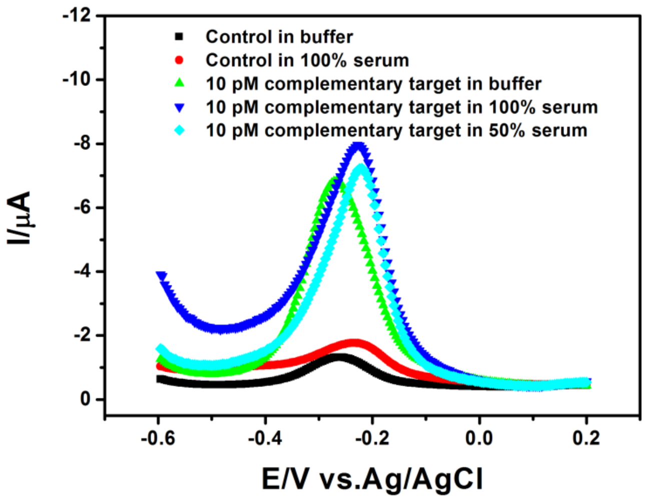

Figure S4. Detection of a DNA oligonucleotide in undiluted serum and 50% serum.

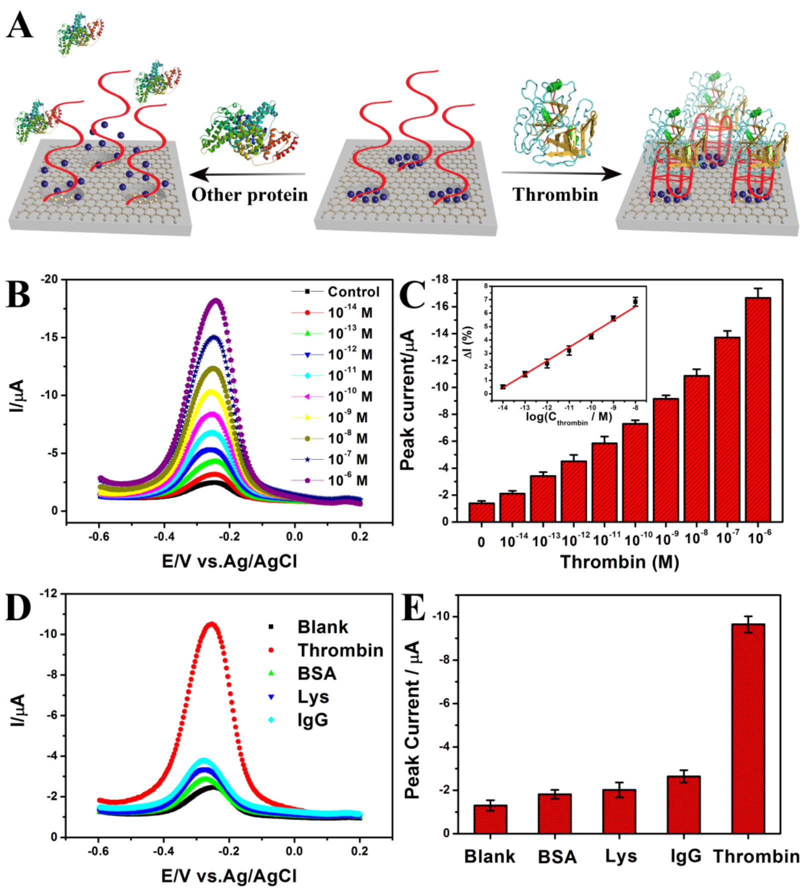

Figure S5. TRE protein detection. (A) Schematic representation of TRE thrombin detection. **(B)** DPVs for thrombin-binding aptamer-modified sensor in response to different concentrations of thrombin: 0, 10^{-14} M, 10^{-13} M, 10^{-12} M, 10^{-11} M, 10^{-10} M, 10^{-9} M, 10^{-8} M, 10^{-7} M and 10^{-6} M. **(C)** Concentration-dependent peak current signal for thrombin detection. Inset: the linear plot. **(D)** The selectivity of thrombin detection using the TRE sensor, the concentration of proteins is 1 nM. **(E)** The value of DPV peak current in the presence of thrombin and other proteins.

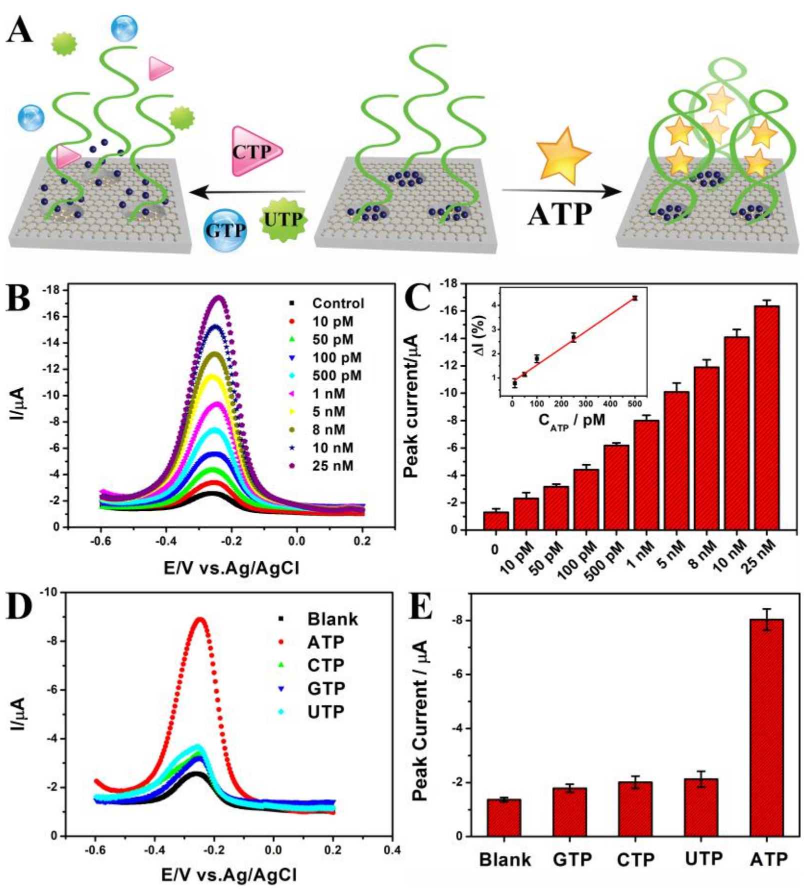

Figure S6. TRE small molecule detection: ATP. (A) Schematic representation of ATP detection by TRE. **(B)** DPVs for ATP-binding aptamer-modified sensor in response to different concentrations of ATP: 0, 10 pM, 50 pM, 100 pM, 1 nM, 5 nM, 8 nM, 10 nM, and 25 nM. **(C)** Concentration-dependent peak current signal for ATP detection. Inset: the linear plot. **(D)** The selectivity of ATP detection using the TRE sensor, the concentration of ATP, CTP, GTP and UTP is 1 nM. **(E)** The value of DPV peak current in the presence of ATP, GTP, CTP and UTP.

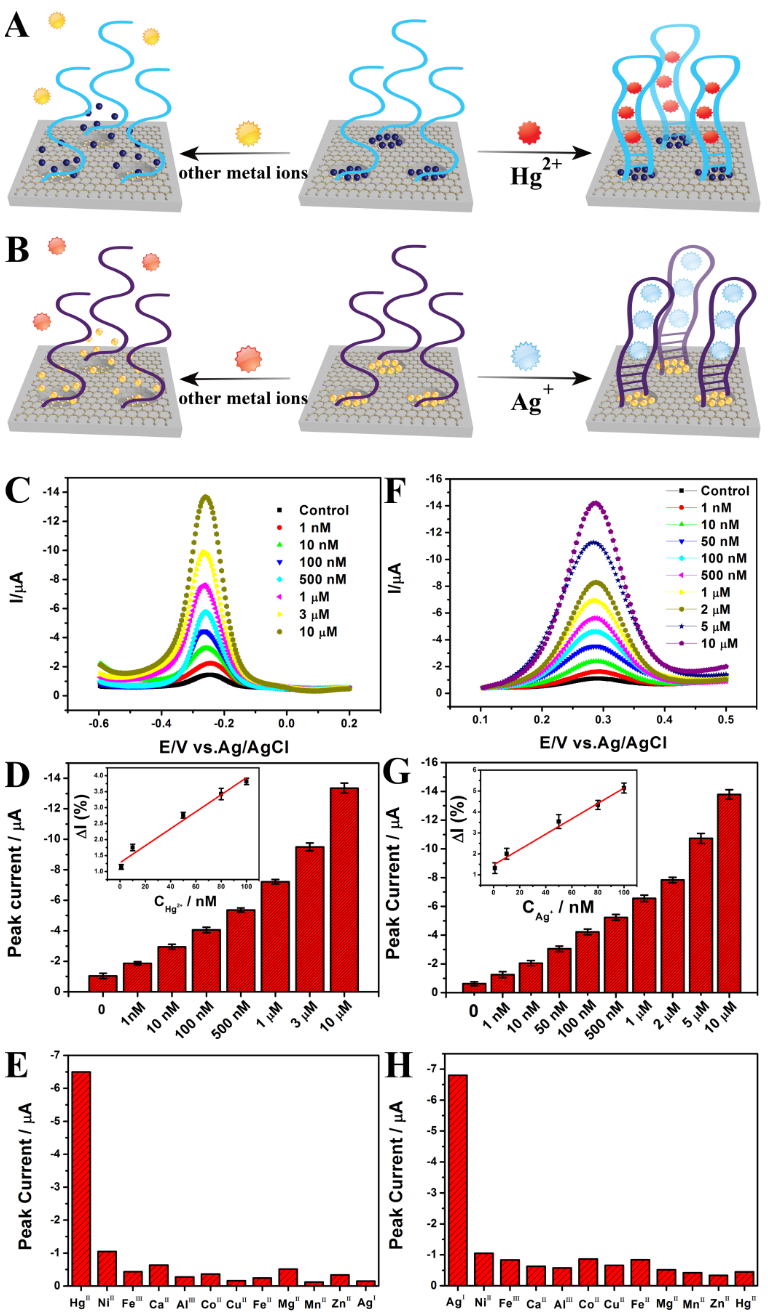

Figure S7*. *TRE small molecule detection: Hg^{2+} and Ag^{+}. (A) Schematic representation of Hg^{2+} detection by TRE with MB as the signal reporter. **(B)** Schematic representation of Ag^{+} detection by TRE with Fc as the signal reporter. **(C)** DPVs for Hg^{2+}-binding DNA probe-modified sensor in response to different concentrations of Hg^{2+}: 0, 1 nM, 10 nM, 100 nM, 500 nM, 1 μM, 3 μM and 10 μM. **(D)** Concentration-dependent peak current signal for Hg^{2+} detection. Inset: the linear plot. **(E)** The selectivity of Hg^{2+} detection using the TRE sensor, the concentration of metal ions is 1 μM. **(F)** DPVs for Ag^{+}-binding DNA probe-modified sensor in response to different concentrations of Ag^{+}: 0, 1 nM, 10 nM, 50 nM, 100 nM, 500 nM, 1 μM, 2 μM, 5 μM and 10 μM. **(D)** Concentration-dependent peak current signal for Ag^{+} detection. Inset: the linear plot. **(E)** The selectivity of Ag^{+} detection using the TRE sensor, the concentration of metal ions is 1 μM.

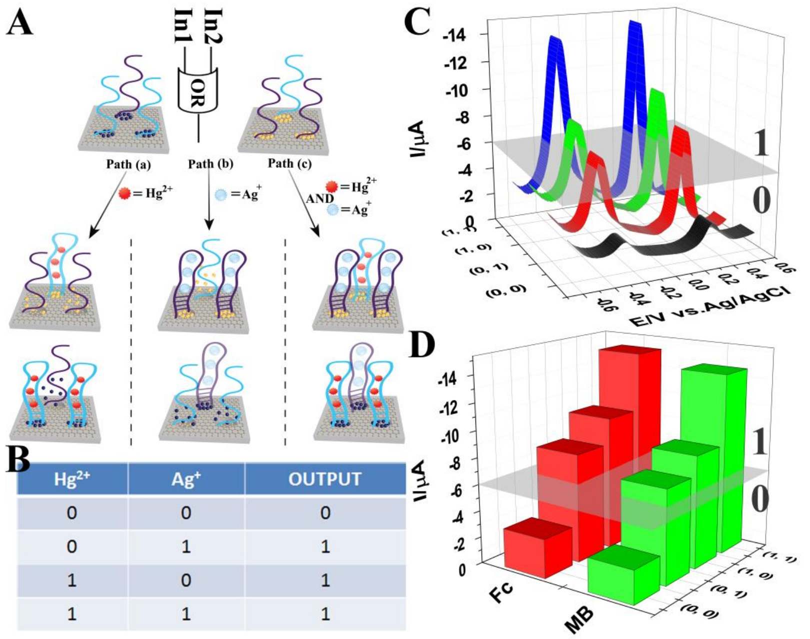

Hg^{2+}	Ag^{+}	OUTPUT
0	0	0
0	1	1
1	0	1
1	1	1

Figure S8. (A) TRE-based OR gate using MSGNs^{MB}-DNA$_{Hg}$$^{2+}$/DNA$_{Ag}$$^{+}$ and MSGNs^{Fc}- DNA$_{Hg}$$^{2+}$/DNA$_{Ag}$$^{+}$; (B) truth table for OR gate; (C) DPVs of a TRE-based OR gate with different combinations of the input: (0,0) no input, (0,1) OR-input-1 (Ag^{+}, 10 μM), (1,0) OR-input-2 (Hg^{2+}, 10 μM), (1,1) OR-input-1 (Ag^{+}, 10 μM) and OR-input-2 (Hg^{2+}, 10 μM); (D) relative current intensities for the OR logic gate.

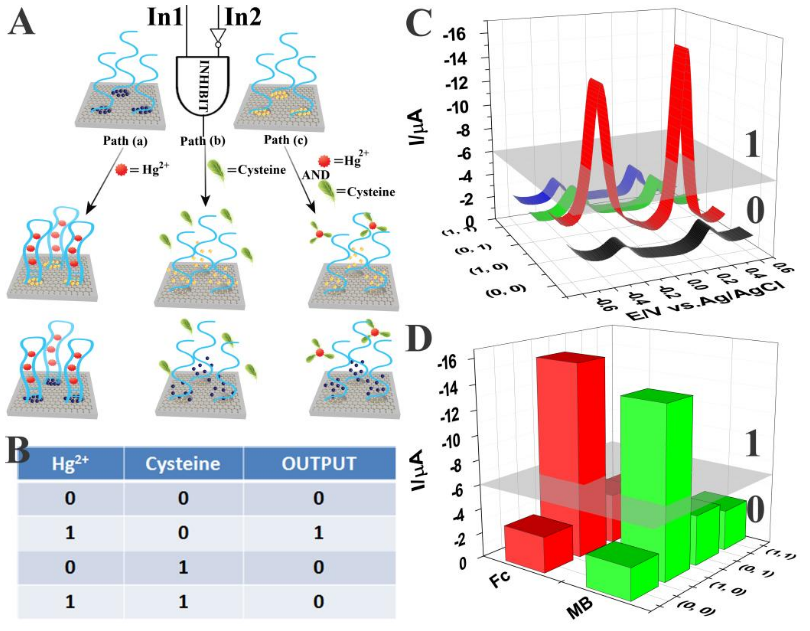

Hg^{2+}	Cysteine	OUTPUT
0	0	0
1	0	1
0	1	0
1	1	0

Figure S9. (A) TRE-based INHIBIT gate using MSGNs^{MB}-DNA$_{Hg}$$^{2+}$ and MSGNs^{Fc}- DNA$_{Hg}$$^{2+}$; (B) truth table for INHIBIT gate; (C) DPVs of a TRE-based INHIBIT gate with different combinations of the input: (0,0) no input, (0,1) INHIBIT-input-1 (Hg^{2+}, 10 μM), (1,0) INHIBIT-input-2 (Cysteine, 20 μM), (1,1)

INHIBIT-input-1 (Hg^{2+}, 10 μM) and INHIBIT-input-2 (Cysteine, 20 μM); (D) relative current intensities for the INHIBIT logic gate.

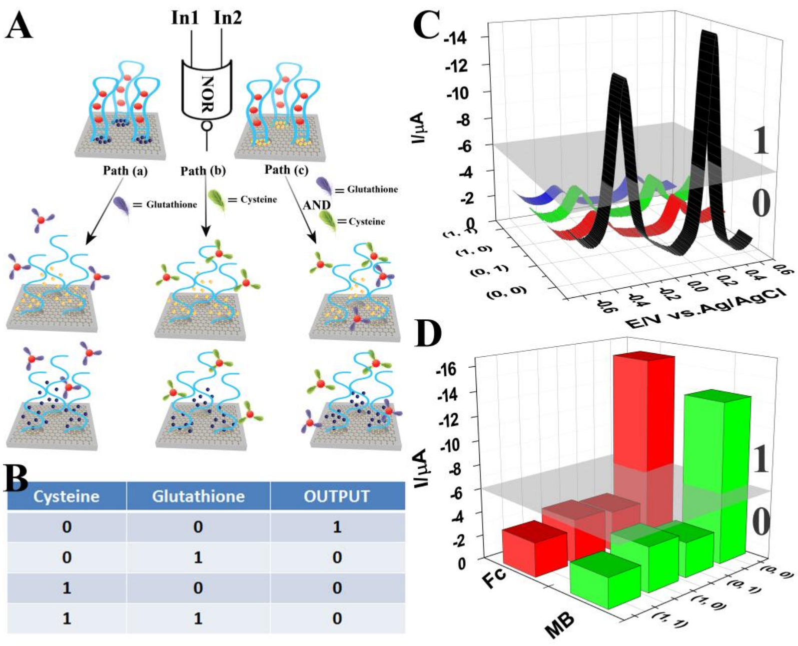

Figure S10. (A) TRE-based NOR gate; (B) truth table for NOR gate; (C) DPVs of a TRE-based NOR gate with different combinations of the input: (0,0) no input, (0,1) NOR-input-1 (Glutathione, 20 μM), (1,0) NOR-input-2 (Cysteine, 20 μM), (1,1) NOR-input-1 (Glutathione, 20 μM) and NOR-input-2 (Cysteine, 20 μM); (D) relative current intensities for the NOR logic gate.

The universal electrochemical detection system, presented here is the first to take the advantages of both graphene and mesoporous silica and achieve sensitivity with all of the major classes of analytes. Compared to the previous work using mesoporous silica nanoparticles as the nanocontainer for amplified fluorescent or colorimetric detection of thrombin, the limit of detection presented in this work is about 4~5 orders of magnitude lower. Furthermore, the sensor developed here also shows the higher sensitivity with all of the major classes of analytes than previous universal detection platform using other techniques, indicating that the combination of graphene and mesoporous silica as the hybrid materials widens the range of capped materials' application and endows them with enormous superiority in electrochemical biosensor fabrication.

Table S1. Comparison of sensors for various analytes detection.

**Materials**	**Method**	**Analyte**	**Limit of detection**
MCM-41 mesoporous silica nanoparticles (1)	Fluorescence	Thrombin	2 nM

Fe_3O_4 nanoparticle encapsulated by mesoporous silica (2)	Colorimetric	Thrombin	0.19 nM
Mesoporous silica nanoparticles (3)	Fluorescence	Hg^{2+}	4 ppb (about 19.9 nM)
Gold nanoparticles and conjugate polyelectrolyte (4)	Colorimetric	DNA	1.25 pM
		Cocaine	10 μM
		Thrombin	10 nM
		Hg^{2+}	50 μM
Invertase/DNA-immobilized magnetic beads (5)	Personal glucose meters	Cocaine	3.4 μM
		Adenosine	18 μM
		Interferon-γ	2.6 nM
		Uranium	9.1 nM
Hemin/G-Quadruplexes and CdSe/ZnS Quantum Dots (6)	Chemiluminescent	Hg^{2+}	10 nM
		ATP	100 nM
		DNA	10 nM
Functionalized graphene and DNA (7)	Colorimetric	Hg^{2+}	8 nM
		DNA	0.5 nM
		Coralyne	0.1 μM
Neutralizer displacement assay-based E-DNA sensor (8)	Electrochemistry	ATP	1 μM
		Cocaine	1 μg/mL
		DNA	100 aM
		E. coli total RNA.	10 pg/μL
		Thrombin	10 fM
		E. coli bacteria	0.15 c.f.u.μL^{-1}
		DNA	1 fM

		Thrombin	10 fM
Graphene-mesoporous silica hybrid nanomaterials (this work)	Electrochemistry	ATP	10 pM
		Hg^{2+}	1 nM
		Ag^{+}	1 nM

References:

1. Oroval, M., Climent, E., Coll, C., Eritja, R., Avino, A., Marcos, M.D., Sancenon, F., Martinez-Manez, R. and Amoros, P. (2013) An aptamer-gated silica mesoporous material for thrombin detection. *Chem. Commun.*, **49**, 5480-5482.

2. Hu, P., Han, L., Zhu, C. and Dong, S.J. (2013) Nanoreactors: a novel biosensing platform for protein assay. *Chem. Commun.*, **49**, 1705-1707.

3. Zhang, Y., Yuan, Q., Chen, T., Zhang, X., Chen, Y. and Tan, W. (2012) DNA-Capped Mesoporous Silica Nanoparticles as an Ion-Responsive Release System to Determine the Presence of Mercury in Aqueous Solutions. *Anal. Chem.*, **84**, 1956-1962.

4. Xia, F., Zuo, X., Yang, R., Xiao, Y., Kang, D., Vallée-Bélisle, A., Gong, X., Yuen, J.D., Hsu, B.B.Y., Heeger, A.J. *et al.* (2010) Colorimetric detection of DNA, small molecules, proteins, and ions using unmodified gold nanoparticles and conjugated polyelectrolytes. *Proc. Natl. Acad. Sci.USA*, **107**, 10837-10841.

5. Xiang, Y. and Lu, Y. (2011) Using personal glucose meters and functional DNA sensors to quantify a variety of analytical targets. *Nat. Chem.*, **3**, 697-703.

6. Freeman, R., Liu, X. and Willner, I. (2011) Chemiluminescent and Chemiluminescence Resonance Energy Transfer (CRET) Detection of DNA, Metal Ions, and Aptamer-Substrate Complexes Using Hemin/G-Quadruplexes and CdSe/ZnS Quantum Dots. *J. Am. Chem. Soc.*, **133**, 11597-11604.

7. Tao, Y., Lin, Y., Ren, J. and Qu, X. (2013) Self-assembled, functionalized graphene and DNA as a universal platform for colorimetric assays. *Biomaterials*, **34**, 4810-4817.

8. Das, J., Cederquist, K.B., Zaragoza, A.A., Lee, P.E., Sargent, E.H. and Kelley, S.O. (2012) An ultrasensitive universal detector based on neutralizer displacement. *Nat.Chem.*, **4**, 642-648.
